# Association of Reactogenicity with Immunogenicity of the ChAdOx1 nCoV-19 Vaccine in Patients Undergoing Hemodialysis

**DOI:** 10.3390/vaccines10081366

**Published:** 2022-08-21

**Authors:** Ting-Yun Lin, Nai-Kuan Hung, Szu-Chun Hung

**Affiliations:** 1 Division of Nephrology, Taipei Tzu Chi Hospital, Buddhist Tzu Chi Medical Foundation, and School of Medicine, Tzu Chi University, Hualien 970374, Taiwan; 2 School of Medicine, National Yang Ming Chiao Tung University, Taipei 112304, Taiwan

**Keywords:** COVID-19, hemodialysis, immunogenicity, reactogenicity, vaccine

## Abstract

The aim of our study was to determine whether local and systemic reactions following SARS-CoV-2 vaccination are predictive of immunogenicity in patients undergoing hemodialysis. We used an established questionnaire to survey 206 hemodialysis patients without prior SARS-CoV-2 infection regarding solicited local (pain, redness, and swelling) and systemic reactions (fatigue, headache, muscle and joint pain, nausea or vomiting, abdominal pain, diarrhea, and fever) within 7 days after receiving 1 dose of the ChAdOx1 nCoV-19 vaccine for SARS-CoV-2. The primary outcome was seroconversion of anti-SARS-CoV-2 IgG (≥50 AU/mL) at 28 days after vaccination. Local and systemic reactions were reported by 80 (38.8%) and 119 (57.8%) patients, respectively. A total of 138 (67.0%) patients developed an antibody response. Responders were younger, had a lower prevalence of coronary artery disease and use of immunosuppressants, and had a higher body mass index and lymphocyte count. In addition, a greater percentage of responders than non-responders reported reactogenicity. In multivariate logistic regression analyses, fever (OR 2.70 [95% CI 1.12–6.50]) and total symptom score (OR 1.33 [95% CI, 1.05–1.68], per one increase) remained strongly associated with a greater humoral response. In conclusion, higher reactogenicity may identify hemodialysis patients who are more responsive to SARS-CoV-2 vaccination.

## 1. Introduction

Patients with end-stage kidney disease (ESKD) who are undergoing maintenance dialysis are highly vulnerable and are at high risk for COVID-19-related morbidity and mortality because of their older age and multiple comorbidities [1,2]. Vaccination is one of the most effective and economical strategies in the fight against SARS-CoV-2. However, the vaccine response is usually attenuated in ESKD patients treated with dialysis due to accelerated immunosenescence induced by chronic inflammation [3]. A recent systematic review and meta-analysis found that patients receiving dialysis had a significantly lower antibody response after the first and second doses of vaccine for SARS-CoV-2 than those not receiving dialysis [4]. Moreover, a great proportion of patients on dialysis were hesitant about seeking COVID-19 vaccination. Patients on dialysis who had vaccine hesitancy were mainly concerned about side effects [5].

Increasing vaccine uptake among patients on dialysis is crucial to mitigating the impact of the COVID-19 pandemic on this vulnerable population. It is undeniable that COVID-19 vaccines can have substantial side effects. Although local and systemic reactions (reactogenicity) are often transient and rarely have serious medical consequences, they may have the most immediate and direct influence on a patient’s perception of the vaccination experience [6]. However, it is highly likely that most of the postvaccination symptoms can be attributed to a short burst of type I interferon (IFN-I) production concomitant with the induction of an effective immune response [7]. Therefore, the side effects of COVID-19 vaccines might be accepted and viewed positively as a prelude to immunization against SARS-CoV-2. Setting expectations regarding what might occur after vaccination may help reduce fears contributing to vaccine hesitancy. However, whether symptoms following COVID-19 vaccination are associated with immunogenicity in hemodialysis patients is unknown.

We hypothesized that local and systemic reactions following vaccination are predictive of a desirable immune response. To elucidate the interplay between reactogenicity and immunogenicity following one dose of COVID-19 vaccine, we examined the frequency of solicited local and systemic side effects after a priming dose of ChAdOx1 nCoV-19, an adenovirus-vectored vaccine expressing the spike protein of SARS-CoV-2, and their association with SARS-CoV-2-specific humoral immunity in patients receiving in-center hemodialysis.

## 2. Methods

### 2.1. Study Design and Participants

To protect this highly vulnerable population from the risk of SARS-CoV-2 infection, the Taiwan Centers for Disease Control and Prevention (CDC) prioritized COVID-19 vaccinations for all patients on dialysis in June 2021. As a result of global vaccine shortages early in the COVID-19 pandemic, patients received homologous ChAdOx1 nCoV-19/ChAdOx1 nCoV-19 prime-boost vaccination, given 12 weeks apart, followed by a third booster dose with a messenger RNA (mRNA) based vaccine (BNT162b2 or mRNA-1273), 12 weeks after the second dose [8].

To investigate the immune response conferred by COVID-19 vaccination, we conducted a prospective observational study of in-center hemodialysis patients who were vaccinated with ChAdOx1 nCoV-19 on June 16 and 17, 2021, and followed until July 15, 2021, at Taipei Tzu Chi Hospital, Taiwan. These vaccines were distributed directly to the dialysis center and were administered at a dose of 5 × 10^10^ viral particles as a single intramuscular injection into the deltoid. Patients who had been clinically stable on hemodialysis for ≥3 months were assessed for eligibility if they were older than 20 years and did not have a history of SARS-CoV-2 infection (either laboratory-confirmed or reported by the participant). Patients were excluded if they were previously vaccinated, refused vaccination, had inadequate dialysis (defined as Kt/V urea < 1.2 or treatment time < 12 h per week), or declined to participate. For all participants, a full medical history was taken, and the medical record was reviewed at the time of enrollment. The diagnosis of diabetes mellitus was based on the current or past use of insulin and/or oral hypoglycemic agents. Hypertension was defined as blood pressure > 140/90 mm Hg or the use of antihypertensive medications. Coronary artery disease (CAD) was diagnosed if there was >50% stenosis in at least one major coronary artery as documented by angiography or a history of myocardial infarction. Participants were observed in the clinic for 30 min after vaccination and were asked to record any adverse events during the 28 day follow-up period. Acetaminophen was permitted if the patient’s temperature was ≥38 °C or for moderate pain. Blood samples were collected on the day of vaccination before the start of dialysis and then again 28 days after vaccination to allow immunogenicity assessments to be made. This study adhered to the Declaration of Helsinki and was approved by the Institutional Review Board of Taipei Tzu Chi Hospital (10-XD-117). Written informed consent was obtained from all the participants.

### 2.2. Reactogenicity Assessments

Data on solicited local (pain, redness, and swelling) and systemic (fatigue, headache, muscle and joint pain, nausea or vomiting, abdominal pain, diarrhea, and fever defined as an ear temperature ≥ 38 °C) reactions that occurred within 7 days after ChAdOx1 nCoV-19 vaccination were collected with the use of a questionnaire established by the Taiwan CDC on day 7 by the participant’s primary nurse. For 28 days following vaccination, data on unsolicited adverse events and severe adverse events were collected.

### 2.3. Immunogenicity Assessments

At day 28, after a priming dose of ChAdOx1 nCoV-19, IgG antibodies to the receptor-binding domain (RBD) of the S1 subunit of the spike protein of SARS-CoV-2 were measured using the AdviseDx SARS-CoV-2 IgG II assay (Abbot Laboratories, Abbott Park, IL, USA), with titers ≥ 50 arbitrary units (AU) per milliliter considered positive [9]. The MeDiPro SARS-CoV-2 Antibody ELISA (Formosa Biomedical Technology, Taipei, Taiwan) was used to detect neutralizing antibodies. Values of ≥12.31 IU/mL (50% neutralizing titer [NT50] ≥ 2.56) were defined as positive humoral responses. This test has 92.2% (95% CI 84.0–96.4%) sensitivity and 93% (95% CI 81.4–97.6%) specificity, according to the manufacturer [10]. The MeDiPro SARS-CoV-2 Antibody ELISA is a surrogate neutralization test, and the results are therefore not actual neutralization titers but surrogate values.

### 2.4. Exposure and Outcome

The main exposure was the presence of reactogenicity within 7 days after vaccination. Individuals were categorized as having or not having any local or systemic reactions. In addition, reactogenicity was graded using a symptom score. For each participant, a score of one was assigned for every local and systemic reaction reported, and then the total symptom score was calculated (maximum 10 points out of 10). The primary outcome was anti-spike IgG antibody seroconversion, defined as antibody levels ≥ 50 AU/mL. Participants were categorized as responders or non-responders based on their anti-spike antibody levels at day 28.

### 2.5. Statistical Analysis

Categorical data are presented as frequencies and percentages and were compared by the chi-square test. Continuous data with or without a normal distribution are presented as the mean ± standard deviation or median (interquartile range) and were compared by Student’s *t* test or the Mann–Whitney U test, respectively. Spearman’s correlation was used to examine the relationship between non-normally distributed datasets. Logistic regression models were used to evaluate the relationship between the exposure and the outcomes of interest. Models were adjusted for patient characteristics, which were selected on the basis of clinical relevance and prior studies and included age, sex, body mass index (BMI), diabetes, CAD, use of immunosuppressants, serum albumin, and lymphocyte count [11,12,13]. Adjusted odds ratios (ORs) with associated 95% CIs were calculated for the group with any local or systemic reaction using the group without any local or systemic reaction, as the reference. In addition, adjusted ORs were calculated using the symptom score as a continuous variable. Two-tailed *p* values < 0.05 were considered statistically significant. All statistical analyses were carried out using the Statistical Package for the Social Sciences software, version 20.0 (SPSS Inc., Chicago, IL, USA).

## 3. Results

### 3.1. Baseline Participant Characteristics 

**Figure 1:**
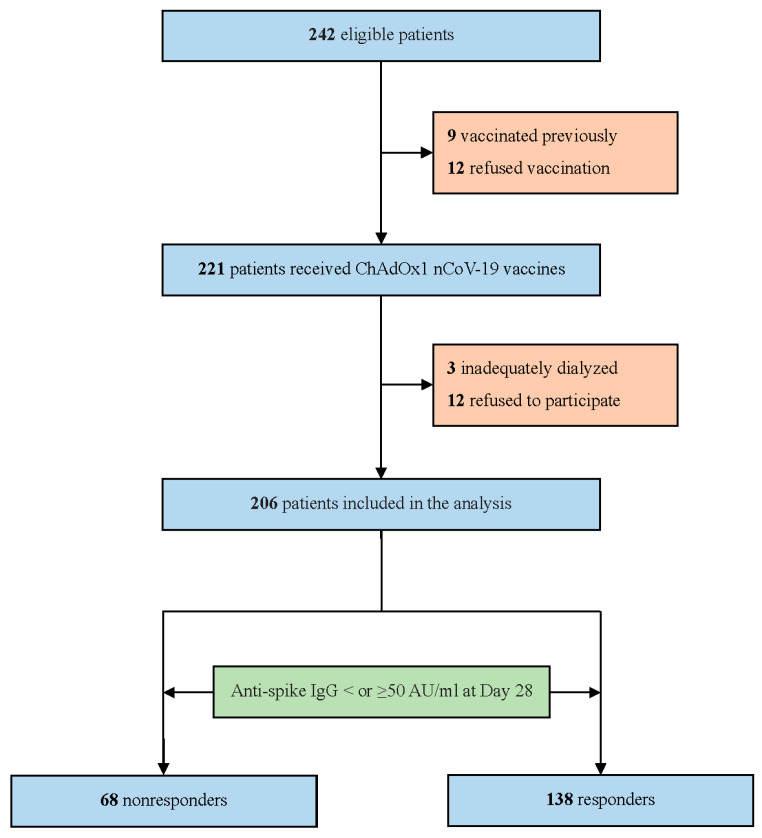
Patient flow diagram.

Of the 242 hemodialysis patients who were screened for eligibility, 36 were excluded. Of those who were excluded, 9 were previously vaccinated, 12 refused vaccination, 3 were dialyzed twice weekly, and 12 declined to participate, leaving a total of 206 patients in the final study population (Figure 1). The baseline characteristics of the participants stratified according to their anti-spike antibody levels into responders (≥50 AU/mL) and non-responders (<50 AU/mL) are presented in Table 1. Overall, the mean age was 67 ± 13 years, 49.5% were women, and 54.4% had diabetes. The mean dialysis vintage was 8.4 ± 5.8 years. Non-responders (*n* = 68) represented 33% of our cohort. Non-responders were generally older, had a lower BMI, and had a higher prevalence of CAD than responders. Non-responders were also more likely to have a higher use of immunosuppressants and had a lower lymphocyte count than responders.

### 3.2. Reactogenicity

**Figure 2:**
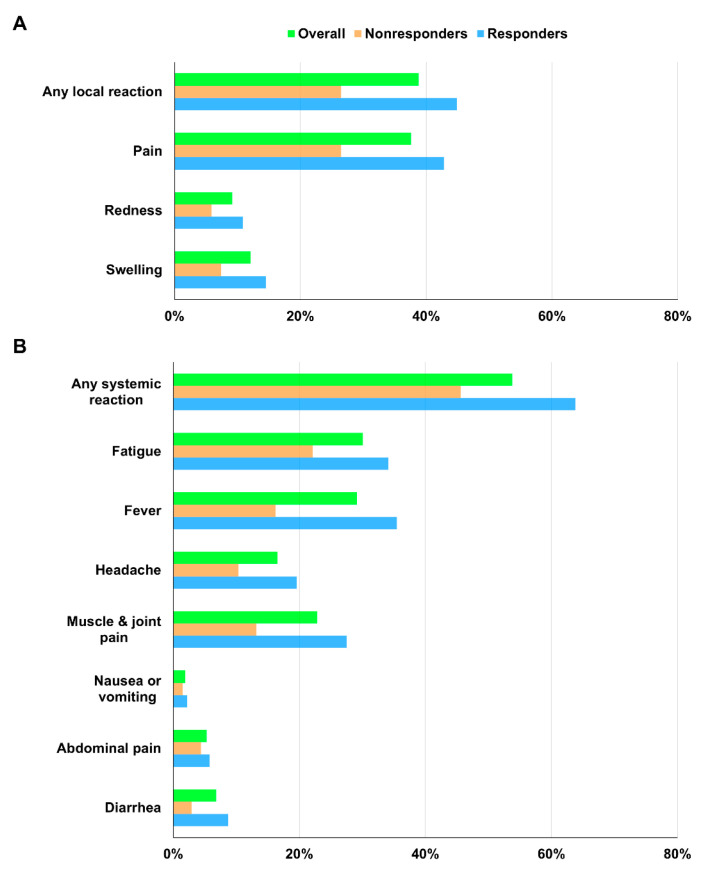
(**A**) Solicited local and (**B**) systemic reactions within 1 week after ChAdOx1 nCoV-19 vaccination according to anti-spike antibody response at day 28.

At least one local symptom was reported by 80 (38.8%) participants, while at least one systemic symptom was reported by 119 (57.8%) participants (Table 2) (Figure 2). Sixty-one participants reported both local and systemic symptoms. These adverse events were generally mild to moderate in severity and typically resolved within 1 or 2 days. The most frequently reported solicited local reactions were injection site pain (37.4%) and swelling (12.1%). The most common solicited systemic reactions were fatigue (30.1%), fever (29.1%), and muscle and joint pain (22.8%). The symptom score frequency and distribution are presented in Supplementary Figure S1. The number of observations decreased as the symptom score increased. No episodes of anaphylaxis or vaccine-induced immune thrombosis with thrombocytopenia were observed during the relatively short safety follow-up period. No serious vaccine-related adverse reactions to ChAdOx1 nCoV-19 occurred.

### 3.3. Immunogenicity

**Figure 3:**
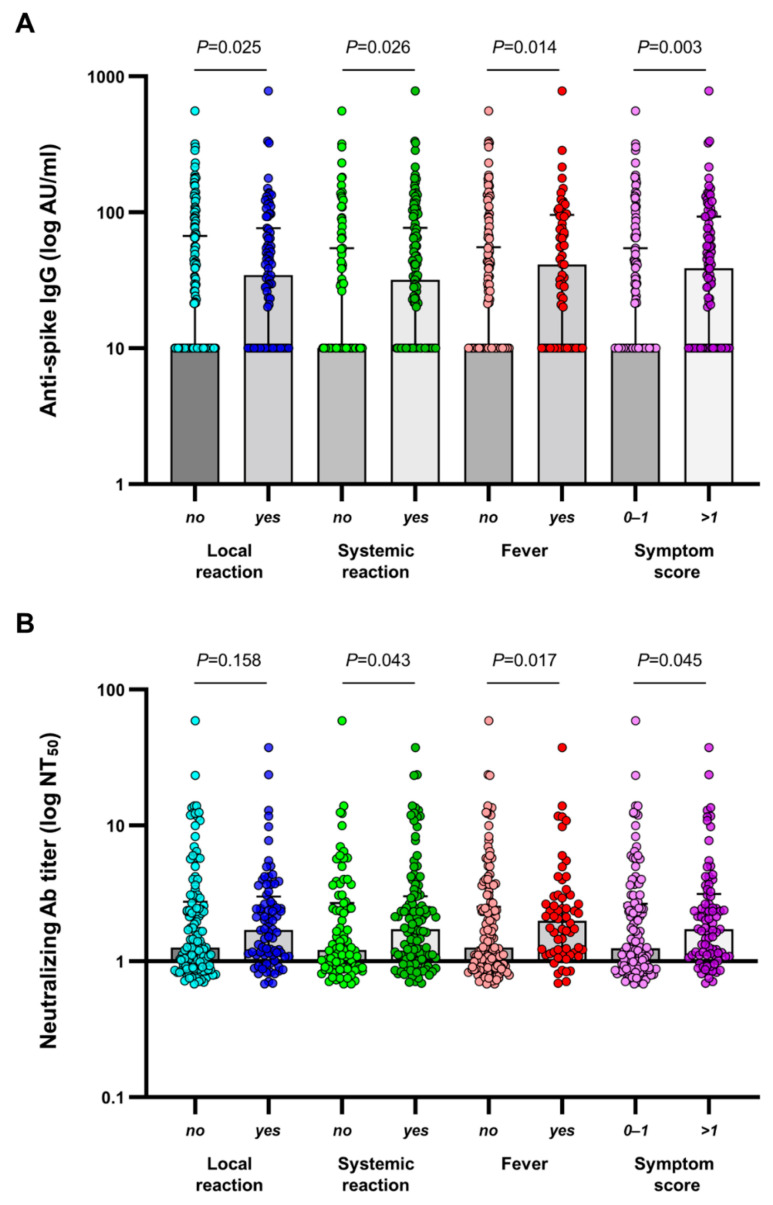
(**A**) SARS-CoV-2 anti-spike IgG antibody concentrations and (**B**) SARS-CoV-2 neutralizing antibody titers at 28 days following the first dose of ChAdOx1 nCoV-19 according to the absence or presence of reactogenicity. The median and interquartile range are shown. IgG values below the lower limit of quantitation were set to 0.5 times the lower limit of quantitation. AU, arbitrary units.

Among the 206 participants in the final analysis, 138 (67.0%) developed a humoral response (anti-spike antibody levels ≥ 50 AU/mL) on day 28 after a single dose of ChAdOx1 nCoV-19. Neutralizing antibody responses against SARS-CoV-2 were detected in 56 (27.2%) patients. Neutralizing antibody titers correlated strongly with anti-spike antibody concentrations (*r* = 0.755, *p* < 0.001) (Supplementary Figure S2). Patients with adverse reactions had significantly higher immunogenicity than those without adverse reactions. The median concentrations of anti-spike antibodies were 243.0 (77.5–538.1) AU/mL and 117.5 (2.8–472.3) AU/mL in patients with and without any local reaction (*p* = 0.025), 224.8 (38.9–542.5) AU/mL and 69.5 (2.6–384.4) AU/mL in patients with and without any systemic reaction (*p* = 0.026), 272.5 (102.3–654.9) AU/mL and 113.1 (1.0–439.2) AU/mL in patients with and without fever (*p* = 0.014), and 272.5 (102.3–654.9) AU/mL and 131.8 (3.0–411.5) AU/mL in patients with symptom score > 1 or ≤1 (*p* = 0.003) (Figure 3A). The distribution of vaccine-induced neutralizing antibodies among patients with or without reactogenicity followed a similar pattern as that seen in anti-spike antibody responses (Figure 3B) (Supplementary Table S1).

### 3.4. Primary Outcome

Local reactions were more common among responders than among non-responders (44.9% and 26.5%, respectively; *p* = 0.011). Similarly, systemic reactions were also more common among responders than among non-responders (63.8% and 45.6%, respectively; *p* = 0.013). There was a significant difference in the total symptom score between non-responders and responders (*p* = 0.002). Multivariate logistic analyses identified age, CAD, the use of immunosuppressive drugs, and lymphocyte count as independent predictors of the primary outcome (Table 3). Each component of reactogenicity and the total symptom score were then entered into the logistic regression models. We found that any local reaction, injection site pain, any systemic reaction, muscle and joint pain, fever, and the total symptom score were predictors of a greater antibody response in univariate analysis, whereas fever (OR, 2.70; 95% CI, 1.12–6.50; *p* = 0.026) and the total symptom score (OR, 1.33; 95% CI, 1.05–1.68; *p* = 0.019 per one increase) remained strongly associated with increased odds of anti-spike antibody seroconversion in multivariate analysis (Table 4).

## 4. Discussion

This study provides the first assessment of the association between reactogenicity and immunogenicity following COVID-19 vaccination in hemodialysis patients. Local and systemic adverse reactions were mostly mild to moderate and self-limiting and were more frequent among responders than non-responders. Although it is a common belief that vaccine side effects are a prerequisite for an effective immune response, previous studies investigating the reactogenicity and immunogenicity of COVID-19 vaccines in non-dialysis patients found inconsistent results [14,15,16]. Our findings suggest that immune responses to the COVID-19 vaccine may be predicted for each hemodialysis patient by analyzing their reactogenicity. Patients undergoing dialysis who experience lower reactogenicity can be considered for enhanced vaccination strategies to reduce the risk of SARS-CoV-2 infection and its complications.

COVID-19 vaccines are effective and essential to control the ongoing pandemic in dialysis patients [17]. However, adverse reactions may contribute to poor uptake [5]. In a prospective observational study among the Taiwanese general population receiving a prime-boost ChAdOx1 nCoV-19 vaccination regimen, the most common solicited local and systemic side effects after the first dose of the standard-dose vaccine were injection site pain (62% of patients aged ≥ 50 years) and fatigue (in 50% of patients aged ≥ 50 years) [18]. In comparison, local and systemic side effects were reported less commonly by hemodialysis patients in the present study (injection site pain in 37% and fatigue in 30% of all the participants). The finding of low rates of adverse events following COVID-19 vaccination in hemodialysis patients is consistent with data from individuals living with multimorbidity. In an online cohort study of 19,586 adults receiving COVID-19 vaccination, the factors most associated with adverse effects were vaccine dose, vaccine brand, younger age, female sex, and having had COVID-19 previously [19]. It is worth noting that older age and comorbid conditions were not associated with higher odds of reporting adverse effects. In a recent study, Lai et al. examined the potential additional risk of adverse events 28 days after the first dose of the COVID-19 vaccine imposed by multimorbidity, defined as ≥2 chronic medical conditions [20]. They found that the association of vaccination with adverse events was not modified by multimorbidity. Since patients with ESKD who are undergoing dialysis are at a high risk of developing poor outcomes if infected with SARS-CoV-2, our results have important implications and should reassure the public about the safety of vaccines with respect to the dialysis population who might be hesitant to receive the vaccine.

In accordance with findings from previous studies that evaluated the COVID-19 vaccine response in hemodialysis patients, we observed that age, cardiovascular disease, the use of immunosuppressive therapy, and lymphocyte count were independent predictors of the primary outcome [11,12,13]. Interestingly, we found reactogenicity to be strongly associated with seroconversion of anti-spike IgG antibodies. Reactogenicity is a physical manifestation of the immune response to vaccination. After entering the body, vaccine antigens are recognized as potential pathogens, resulting in the synthesis and release of pyrogenic cytokines into the systemic circulation. These innate immune responses are crucial for triggering strong antigen-specific adaptive immune responses necessary for protection against disease, but these same inflammatory events may also account for the development of side effects in the vaccinated individual [21]. These transient adverse reactions are of minor clinical relevance because they cause no lasting harm but are often misperceived as a greater risk than infectious disease [22]. If a vaccine’s reactogenicity is perceived as an indicator of successful immunization outcomes, a person’s willingness to be vaccinated can be improved.

Although clinical outcomes are the most appropriate method for evaluating vaccine response, data on the efficacy of COVID-19 vaccines in dialysis patients are limited because these patients have been largely excluded from clinical trials. One available approach is to examine how a vaccine elicits humoral and cellular responses. Higher levels of binding or neutralizing antibodies have been shown to correlate with a reduced risk of symptomatic COVID-19 infection [23]. However, antibody testing after vaccination is not recommended by the US CDC to assess individuals’ responses to immunization. To support decision-making regarding boosters in hemodialysis patients who have received a priming dose of COVID-19 vaccine, postvaccination symptoms may help identify those who develop immune responses to SARS-CoV-2. This would enable us to personalize the COVID-19 vaccination protocol.

Limitations of this study include the relatively small sample size and the short duration of follow-up. We defined the primary outcome as seroconversion of anti-spike antibodies at day 28 because humoral responses to SARS-CoV-2 spike protein peaked by day 28 post prime dose [24]. Second, as with any observational study, there is the possibility of residual confounding. Third, the timing of survey collection may have introduced recall bias and affected symptom reporting. Finally, these results are from a mainly Asian population, which may limit the generalizability of our findings. In addition, immunological data from an adenovirus-vectored vaccine may not be applicable for all vaccines.

## 5. Conclusions

The ChAdOx1 nCoV-19 vaccine was safe and well tolerated, with a lower reactogenicity profile in the hemodialysis population than in the healthy population. Immunization with a priming dose of ChAdOx1 nCoV-19 results in the induction of humoral immune responses against SARS-CoV-2 in two-thirds of hemodialysis patients, with a greater percentage of responders than non-responders reporting reactogenicity. Vaccine-associated side effects may contribute to poor uptake, but the results of our study showed that reactogenicity is also a predictive sign of a desirable immune response. These findings can have a positive effect on vaccine acceptance by providing our patients on dialysis with higher perceived benefits from the COVID-19 vaccine.

## Figures and Tables

**Table 1:** Baseline characteristics of hemodialysis patients according to their anti-spike IgG antibody response at day 28.

Variables	All (*n* = 206)	Nonresponders (*n* = 68)	Responders (*n* = 138)	*p* Value
Demographic data				
Age (year)	66.9 ± 12.5	72.5 ± 10.9	64.1 ± 12.4	<0.001
Male sex, *n* (%)	104 (50.5%)	37 (54.4%)	67 (48.6%)	0.429
Smoking history, *n* (%)	39 (18.9%)	15 (22.1%)	24 (17.4%)	0.421
Dialysis vintage (year)	8.4 ± 5.8	9.3 ± 6.6	7.9 ± 5.3	0.102
Kt/V	1.7 ± 0.3	1.7 ± 0.3	1.7 ± 0.2	0.391
URR (%)	75.9 ± 5.4	76.2 ± 6.7	75.8 ± 4.7	0.618
nPCR (g/kg/day)	1.07 (0.92–1.24)	1.05 (0.91–1.16)	1.09 (0.93–1.27)	0.106
Body mass index (kg/m^2^)	23.4 ± 3.8	22.6 ± 4.1	23.8 ± 3.6	0.032
Diabetes mellitus, *n* (%)	112 (54.4%)	42 (61.8%)	70 (50.7%)	0.135
Hypertension, *n* (%)	186 (90.3%)	62 (91.2%)	124 (89.9%)	0.763
CAD, *n* (%)	52 (25.2%)	27 (39.7%)	25 (18.1%)	0.001
Stroke, *n* (%)	8 (3.9%)	4 (5.9%)	4 (2.9%)	0.297
Malignancy, *n* (%)	24 (11.7%)	10 (14.7%)	14 (10.1%)	0.337
Use of IS, *n* (%)	5 (2.4%)	4 (5.9%)	1 (0.7%)	0.024
Laboratory data				
Albumin (g/dL)	3.8 (3.6–4.0)	3.7 (3.5–4.0)	3.9 (3.7–4.0)	0.073
Fasting glucose (mg/dL)	143 (114–192)	151 (125–201)	138 (114–191)	0.221
Lymphocyte (×10^9^/L)	1.1 (0.9–1.4)	1.0 (0.7–1.2)	1.2 (1.0–1.5)	<0.001
Hemoglobin (g/dL)	10.4 (9.5–11.0)	10.4 (9.3–11.2)	10.4 (9.6–11.0)	0.707
Ferritin (ng/mL)	461 (250–644)	443 (279–645)	474 (225–644)	0.868
Calcium (mg/dL)	9.4 (8.8–10.0)	9.4 (8.8–10.0)	9.4 (8.9–10.1)	0.432
Phosphate (mg/dL)	4.4 (3.6–5.3)	4.2 (3.6–5.6)	4.5 (3.7–5.2)	0.691
iPTH (pg/mL)	326 (140–593)	348 (126–600)	326 (141–593)	0.931

CAD = coronary artery disease; iPTH = intact parathyroid hormone; IS = immunosuppressant; nPCR = normalized protein catabolic rate; URR = urea reduction ratio.

**Table 2:** Solicited local and systemic reactions to the ChAdOx1 nCoV-19 vaccine reported 0 to 7 days after vaccination in hemodialysis patients according to their anti-spike IgG antibody response at day 28.

Variables	All (*n* = 206)	Nonresponders (*n* = 68)	Responders (*n* = 138)	*p* Value
Any local reaction, *n* (%)	80 (38.8%)	18 (26.5%)	62 (44.9%)	0.011
Pain, *n* (%)	77 (37.4%)	18 (26.5%))	59 (42.8%)	0.023
Redness, *n* (%)	19 (9.2%)	4 (5.9%)	15 (10.9%)	0.245
Swelling, *n* (%)	25 (12.1%)	5 (7.4%)	20 (14.5%)	0.140
Any systemic reaction, *n* (%)	119 (57.8%)	31 (45.6%)	88 (63.8%)	0.013
Fatigue, *n* (%)	62 (30.1%)	15 (22.1%)	47 (34.1%)	0.077
Headache, *n* (%)	34 (16.5%)	7 (10.3%)	27 (19.6%)	0.092
Muscle and joint pain, *n* (%)	47 (22.8%)	9 (13.2%)	38 (27.5%)	0.021
Nausea or vomiting, *n* (%)	4 (1.9%)	1 (1.5%)	3(2.2%)	0.073
Abdominal pain, *n* (%)	11 (5.3%)	3 (4.4%)	8 (5.8%)	0.677
Diarrhea, *n* (%)	14 (6.8%)	2 (2.9%)	12 (8.7%)	0.123
Fever, *n* (%)	60 (29.1%)	11 (16.2%)	49 (35.5%)	0.004
Symptom score ^a^	1.0 (0.0–3.0)	1.0 (0.0–1.0)	1.0 (0.0–3.0)	0.002

^a^ A score of one was awarded for every local and systemic reaction reported, and then the total symptom score was calculated for each participant (maximum 10 points out of 10).

**Table 3:** Baseline characteristics as determinants of the anti-spike IgG antibody response to SARS-CoV-2 vaccination.

Variables	Univariate		Multivariate	
	OR (95% CI)	*p* Value	OR (95% CI)	*p* Value
Age (year)	0.94 (0.92–0.97)	<0.001	0.93 (0.90–0.97)	<0.001
Male sex	0.79 (0.44–1.42)	0.429	0.57 (0.27–1.20)	0.139
Body mass index (kg/m^2^)	1.09 (1.01–1.18)	0.033	1.00 (0.91–1.10)	0.963
Diabetes mellitus	0.64 (0.35–1.15)	0.136	0.67 (0.32–1.39)	0.279
CAD	0.34 (0.18–0.64)	0.001	0.26 (0.11–0.59)	0.001
Use of IS	0.12 (0.01–1.07)	0.057	0.02 (0.00–0.24)	0.002
Albumin (g/dL)	3.19 (1.14–8.96)	0.027	3.03 (0.78–11.81)	0.110
Lymphocyte (×10^9^/L)	5.21 (2.31–11.71)	<0.001	4.14 (1.69–10.17)	0.002

CAD = coronary artery disease; IS = immunosuppressant; OR = odds ratio.

**Table 4:** Solicited local and systemic reactions as determinants of anti-spike IgG antibody response to SARS-CoV-2 vaccination.

Variables	Univariate		Multivariate	
	OR (95% CI)	*p* Value	OR (95% CI)	*p* Value
Local reaction	2.27 (1.20–4.28)	0.012	2.11 (0.98–4.54)	0.056
Pain	2.08 (1.10–3.92)	0.024	1.92 (0.89–4.13)	0.096
Redness	1.95 (0.62–6.12)	0.252	2.48 (0.61–10.10)	0.205
Swelling	2.14 (0.77–5.96)	0.147	1.72 (0.49–6.04)	0.399
Systemic reaction	2.10 (1.16–3.79)	0.014	1.60 (0.79–3.23)	0.193
Fatigue	1.83 (0.93–3.58)	0.080	1.74 (0.79–3.85)	0.169
Headache	2.12 (0.87–5.15)	0.097	1.32 (0.46–3.85)	0.606
Muscle and joint pain	2.49 (1.13–5.51)	0.024	1.43 (0.54–3.75)	0.471
Nausea or vomiting	1.49 (0.15–14.59)	0.732	2.54 (0.11–58.71)	0.564
Abdominal pain	1.33 (0.34–5.19)	0.678	1.78 (0.34–9.31)	0.494
Diarrhea	3.14 (0.68–14.46)	0.141	4.03 (0.59–27.35)	0.154
Fever	2.85 (1.37–5.94)	0.005	2.70 (1.12–6.50)	0.026
Symptom score (per one increase)	1.37 (1.13–1.66)	0.002	1.33 (1.05–1.68)	0.019

OR = odds ratio. Adjusted for age, sex, body mass index, diabetes mellitus, coronary artery disease, use of immunosuppressant, albumin, and lymphocyte count.

## Data Availability

Data supporting the findings in this manuscript are available from the corresponding author upon request.

## Supplementary Materials

The following supporting information can be downloaded at: https://www.mdpi.com/article/10.3390/vaccines10081366/s1; Supplementary Table S1. Immune responses (median and interquartile range) in hemodialysis patients according to reactogenicity. Supplementary Figure S1. Distribution of the symptom score. Supplementary Figure S2. Correlations between SARS-CoV-2 anti-spike IgG antibody concentrations and neutralizing antibody titers.
