# Supplementary material for: Association of Reactogenicity with Immunogenicity of the ChAdOx1 nCoV-19 Vaccine in Patients Undergoing Hemodialysis
Source: Vaccines (Basel). 2022 Aug 21;10(8):1366. doi: 10.3390/vaccines10081366 (PMC9412992; doi:10.3390/vaccines10081366)

Supplementary Table of Contents

Supplementary Table S1. Immune responses (median and interquartile range) in hemodialysis patients according to reactogenicity

Supplementary Figure S1. Distribution of the symptom score.

Supplementary Figure S2. Correlations between SARS-CoV-2 anti-spike IgG antibody concentrations and neutralizing antibody titers.

Supplementary Table S1. Immune responses (median and interquartile range) in hemodialysis patients according to reactogenicity

**Reactogenicity**	**Yes**	**No**	***P* value**
**Local reaction**	*n*=80	*n*=126	
Anti-spike antibody (AU/mL)	243.0 (77.5–538.1)	117.5 (2.8–472.3)	0.025
Neutralizing antibody (NT_{50})	1.71 (1.11–3.00)	1.26 (0.91–2.62)	0.158
**Systemic reaction**	*n*=119	*n*=87	
Anti-spike antibody (AU/mL)	224.8 (38.9–542.5)	69.5 (2.6–384.4)	0.026
Neutralizing antibody (NT_{50})	1.72 (1.09–3.01)	1.21 (0.90–2.52)	0.043
**Fever**	*n*=60	*n*=146	
Anti-spike antibody (AU/mL)	267.4 (108.6–661.2)	131.8 (3.0–411.5)	0.014
Neutralizing antibody (NT_{50})	1.99 (1.16–3.07)	1.26 (0.91–2.47)	0.017
**Symptom score >1**	*n*=82	*n*=124	
Anti-spike antibody (AU/mL)	272.5 (102.3–654.9)	113.1 (1.0–439.2)	0.003
Neutralizing antibody (NT_{50})	1.73 (1.11–3.14)	1.24 (0.90–2.56)	0.045

AU, arbitrary unit; NT_{50}, 50% neutralizing titer.

Supplementary Figure S1. Distribution of the symptom score.

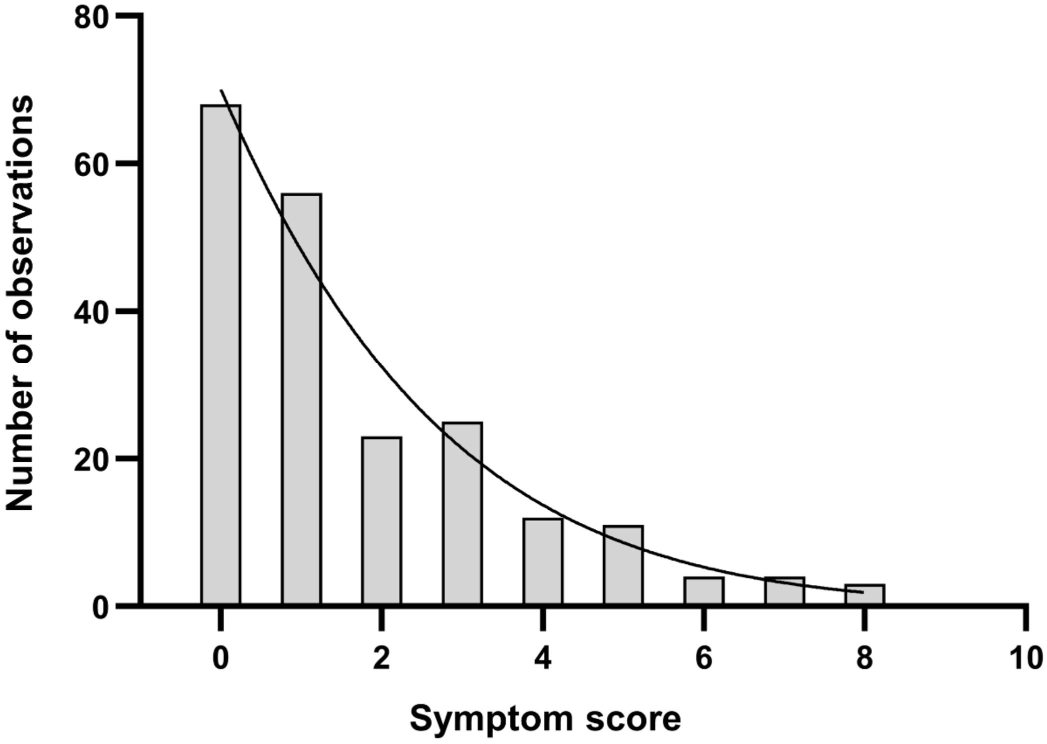

Supplementary Figure S2. Correlations between SARS-CoV-2 anti-spike IgG antibody concentrations and neutralizing antibody titers.

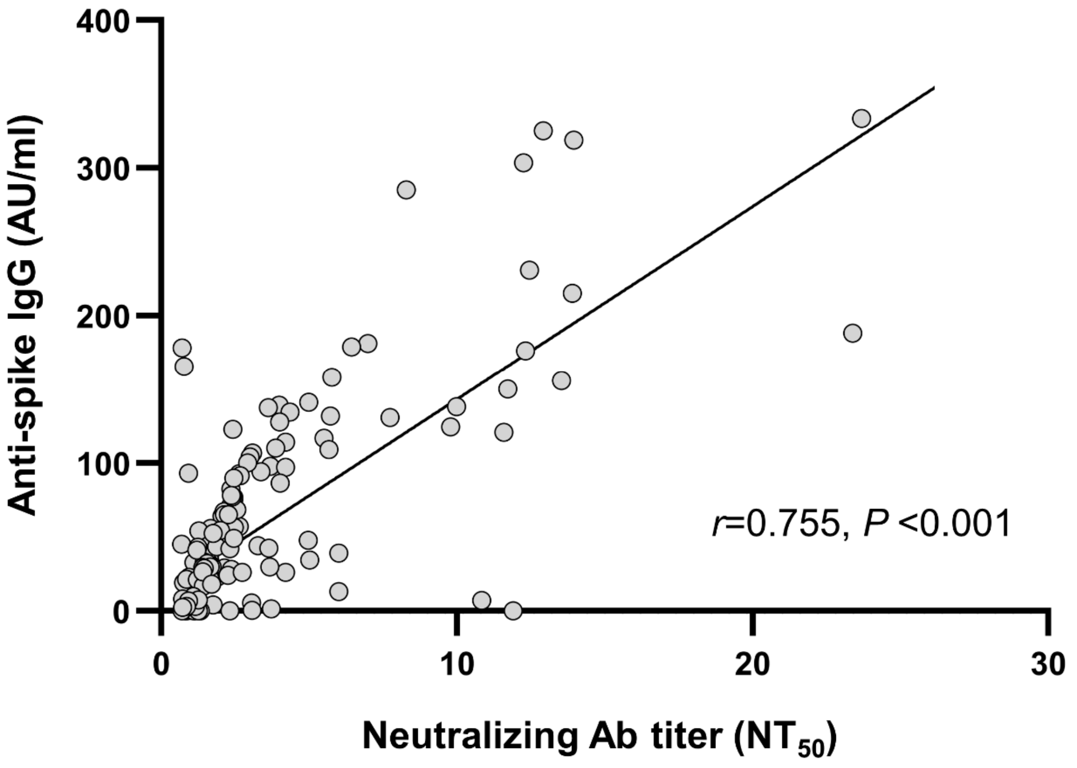

Supplement: Supplementary file 1 [file vaccines-10-01366-s001.zip › vaccines-1864093-supplementary.pdf]
